# Case report: Cochlear implantation was effective for progressive bilateral severe hearing loss associated with Kawasaki disease

**DOI:** 10.3389/fped.2023.1199240

**Published:** 2023-08-10

**Authors:** Daichi Murakami, Takahito Kimura, Masamitsu Kono, Akihiro Sakai, Tomohiro Suenaga, Masanobu Hiraoka, Hideki Sakatani, Makiko Ohtani, Hiroyuki Suzuki, Daisuke Tokuhara, Muneki Hotomi

**Affiliations:** ^1^ Department of Otorhinolaryngology-Head and Neck Surgery, Wakayama Medical University, Wakayama, Japan; ^2^ Sakai ENT Clinic, Kinokawa, Japan; ^3^ Department of Pediatrics, Wakayama Medical University, Wakayama, Japan; ^4^ Department of Pediatrics, Wakayama Tsukushi Medical and Welfare Center, Iwade, Japan

**Keywords:** Kawasaki disease, hearing loss, cochlear implantation, speech development, systemic vasculitis

## Abstract

Sensorineural hearing loss associated with Kawasaki disease has been increasingly reported, but its etiology remains unclear. Most reported cases of sensorineural hearing loss associated with Kawasaki disease have been mild and reversible during acute or subacute phases. However, bilateral severe hearing loss as a complication of Kawasaki disease can cause delays in cognitive and speech development. A 4-year-old Japanese boy treated for Kawasaki disease had right-side moderate and left-side profound sensorineural hearing loss on the 141st day after onset of Kawasaki disease. Despite systemic steroid pulse treatment, hearing loss remained in both sides. After the recurrence of Kawasaki disease, hearing on the right side progressively worsened, meaning there was now severe hearing loss on both sides. Left cochlear implantation performed on the 1065th day after the onset of Kawasaki disease improved the patient's hearing and his ability to communicate. Sensorineural hearing loss associated with Kawasaki disease may progress over a long period and cause bilateral severe hearing loss, although past reports showed occurrence during acute or subacute phases. The clinical course of our patient suggests that intense inflammation caused by Kawasaki disease could be related to prolonged hearing loss. Cochlear implantation seems to be effective for sensorineural hearing loss associated with Kawasaki disease.

## 1. Introduction

Kawasaki disease (KD), which was first described in Japan in 1967 (1), is a mucocutaneous lymph node syndrome presenting polymorphus exanthema, changes in peripheral extremities, bilateral nonexudative conjunctival injection, changes in the oropharyngeal mucosa and acute nonsuppurative cervical lymphadenopathy (2). Besides these major clinical symptoms, some patients have presented unusual otolaryngological symptoms (3, 4). Sensorineural hearing loss (SNHL) associated with KD has been increasingly reported (3, 5–8). We also have reported some cases of SNHL potentially associated with KD in Japan (9). Approximately 30% of all patients with KD are affected by some degree of SNHL (3, 5, 6). SNHL may therefore be more common than most other complications of KD, including coronary artery aneurysms (3).

The clinical course of SNHL associated with KD varies between patients. Although most SNHL associated with KD usually develops within the first 30 days of disease onset and is reversible, 5.5%–14% of patients have persistent SNHL (3, 5, 6). Progression over a long term seems to be rare and results in underestimation of its association with KD. Although SNHL associated with KD is usually mild hearing loss (5, 7, 8), there are a few reports of patients that developed severe hearing loss (10–12). Most cases of KD occur in children less than five years of age (6), so poor prognosis of bilateral SNHL associated with KD can cause delays in cognitive and speech development (13). The etiology of SNHL remains unclear, so there is no established treatment. Although treatment with intravenous immunoglobulins (IVIG), aspirin and systemic steroids are therapeutic choices in the acute phase (14), there are no effective therapeutic interventions for persistent SNHL.

Herein, we describe the case of a 4-year-old Japanese boy with KD with bilateral severe SNHL that progressed over 2 years. This report shows the efficacy of cochlear implantation for SNHL associated with KD.

## 2. Case description

**Figure 1:**
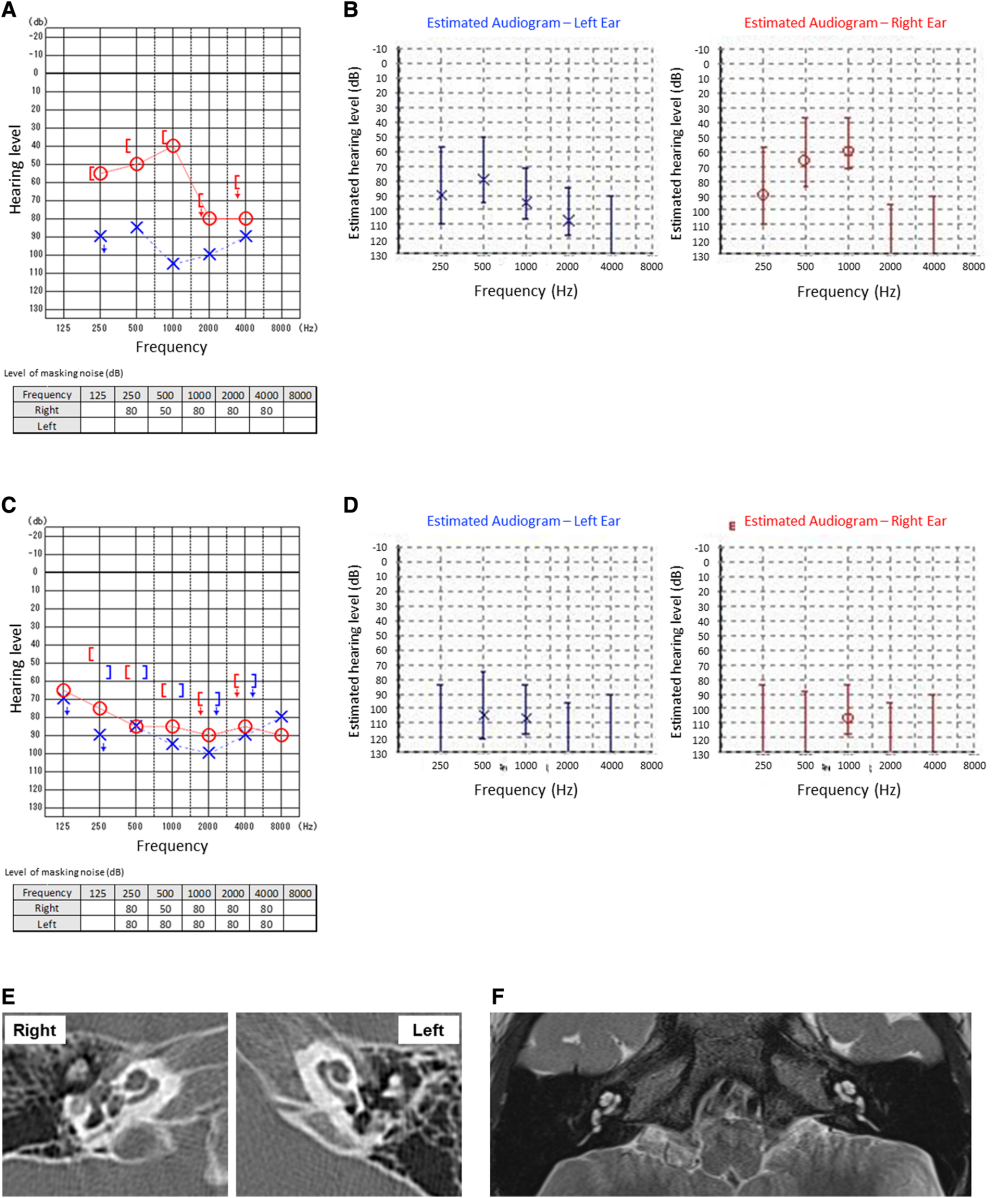
Auditory tests at the first visit (**A**,**B**) and after the relapse of Kawasaki disease (**C**,**D**). Pure tone audiometry tests (**A**,**C**) and auditory steady-state response tests (**B**,**D**) were examined. At the first visit, the patient was unable to complete the left pure tone bone conduction audiometry due to his age (**A**). CT (**E**) and MRI (**F**) showed no significant findings including labyrinthine and ossifications in the cochlea.

The patient is 4-year-old Japanese boy who had no notable past clinical history or family history but developed KD. From the fourth day after onset, he began treatment with IVIG and oral aspirin (30 mg/kg/day). However, he was persistently febrile and was treated with cyclosporin (in the range of 6.5–9.7 mg/kg/day for a total of 10 days) and subsequent oral prednisolone (gradually reduced from 0.8 mg/kg) in the pediatrics department. Blood examinations showed the persistent elevation of white blood cell (WBC) count and C-reactive protein (CRP), anemia and thrombocytosis during the treatment for KD. Blood concentrations of cyclosporine were also monitored to be within therapeutic range but not ototoxic levels as maximum 80.4 ng/ml. After the first treatment, he had no complications, including coronary aneurysm or the need for continuous medications. From the 130th day after onset of KD, his family noticed that he had impaired hearing. On the 141st day after onset of KD, he was presented to the otorhinolaryngology-head and neck surgery department. The pure tone audiometry and auditory steady-state response (ASSR) test showed right-side moderate and left-side severe sensory hearing loss (Figures 1A,B). The tympanic membrane showed no findings of otitis media, such as redness or middle ear effusion. There were no significant findings including labyrinthine ossifications in the cochlea in computed tomography (CT) of the temporal bone or magnetic resonance imaging (MRI) of the inner ear. Otoacoustic emission test was not performed. Blood examinations were unremarkable, showing no elevation of WBC count or CRP. We immediately started intravenous prednisolone treatment (gradually reduced from 0.6 mg/kg), but there was no significant improvement of hearing loss on either side. Given so young age of the patient that he could not keep him still during the procedure, we could not perform salvage intratympanic injections of steroids. An air conduction hearing aid was applied to the patient's right ear. No inspectable gene variants relating to hearing loss were identified by a commercially available screening test with next generation sequence including 19 genes and 154 variants (BML, INC., Tokyo, Japan).

**Figure 2:**
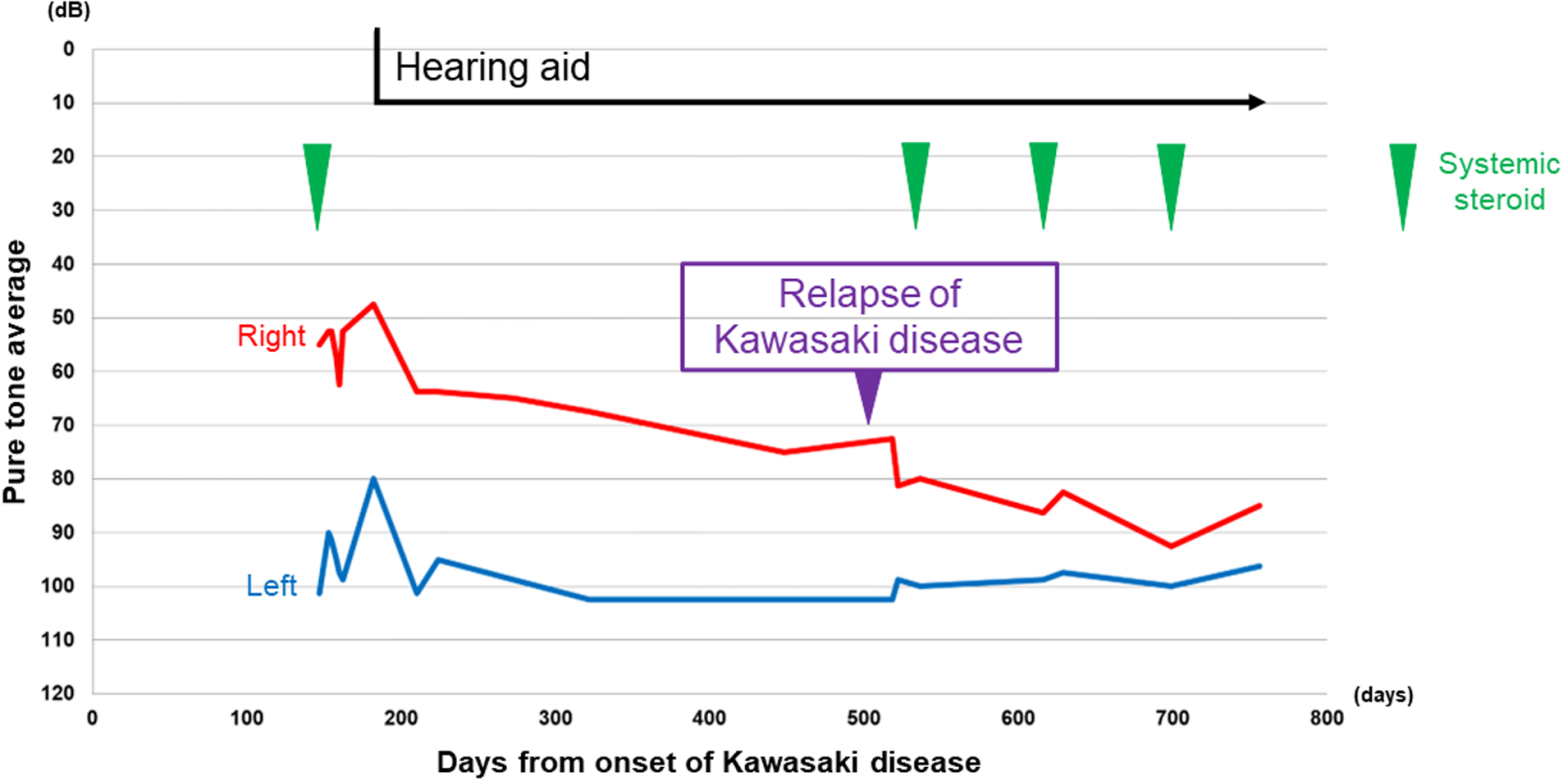
Time course of the hearing level by pure tone audiometry. The average was calculated by the following formula: (*T*_500_ + *T*_1,000_ × 2 + *T*_2,000_)/4. *T*_500_, *T*_1,000_, and *T*_2,000_ indicate the level of air conduction threshold of 500, 1,000, and 2,000, respectively. Red line: right pure tone average, blue line: left pure tone average. Vertical axis: hearing level in decibels, horizontal axis: days since the onset of Kawasaki disease. Green arrowhead: timing of systemic steroid.

On the 510th day after the first onset, there was relapse of KD. He recovered after immediate IVIG treatment, but his right-side SNHL worsened. After treatment for KD, systemic steroid treatment for right-side SNHL was reintroduced, but there was no improvement of hearing loss, rather, there was progressive deterioration. Blood examinations showed persistent mild anemia and thrombocytosis during the progression of hearing loss, although there was no elevation of WBC count. Finally, the patient developed severe hearing loss on the right side (Figures 1C,D). The time course of hearing level of both sides by pure tone audiometry is shown in Figure 2. His mental state subsequently became very unstable due to a lack of auditory communication, even when using hearing aids on both sides.

**Figure 3:**
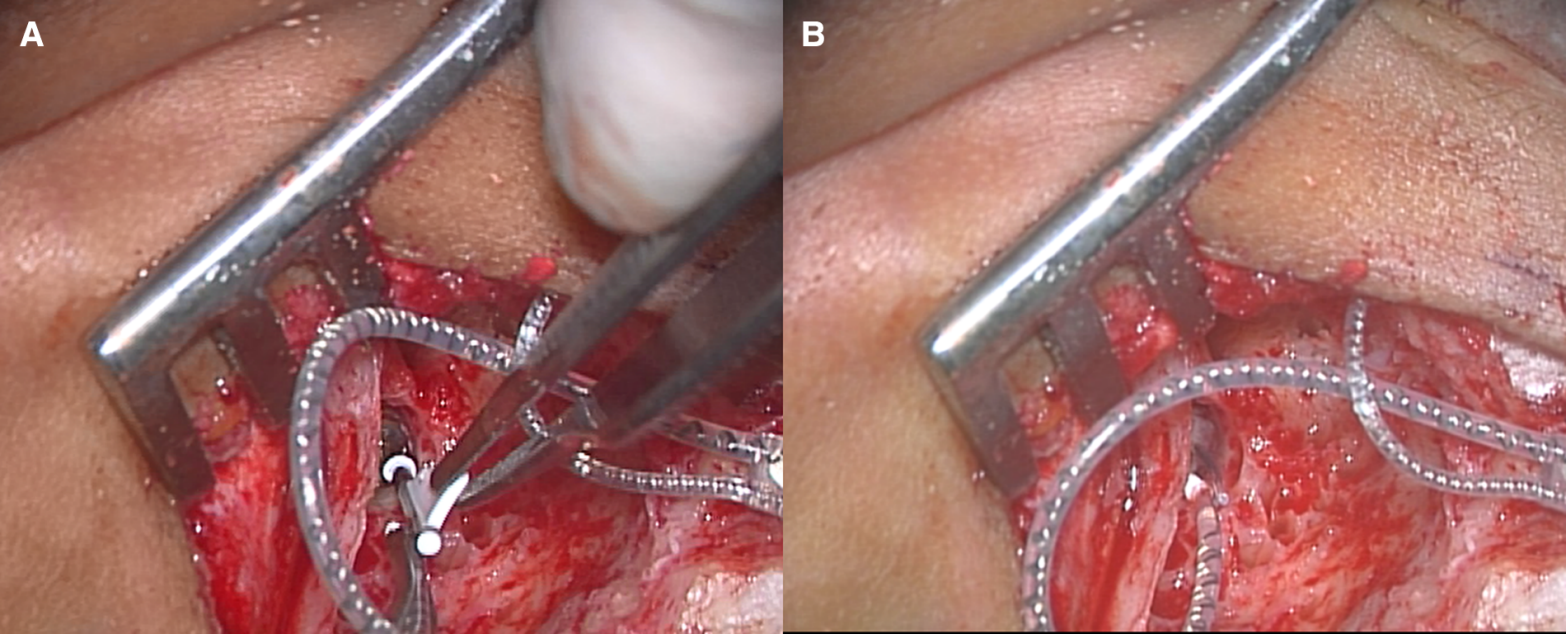
Left cochlear implantation. (**A**) The sheath of slim modiolar electrode was inserted into the round window. (**B**) After complete insertion of the electrode through the round window, the sheath was removed.

**Figure 4:**
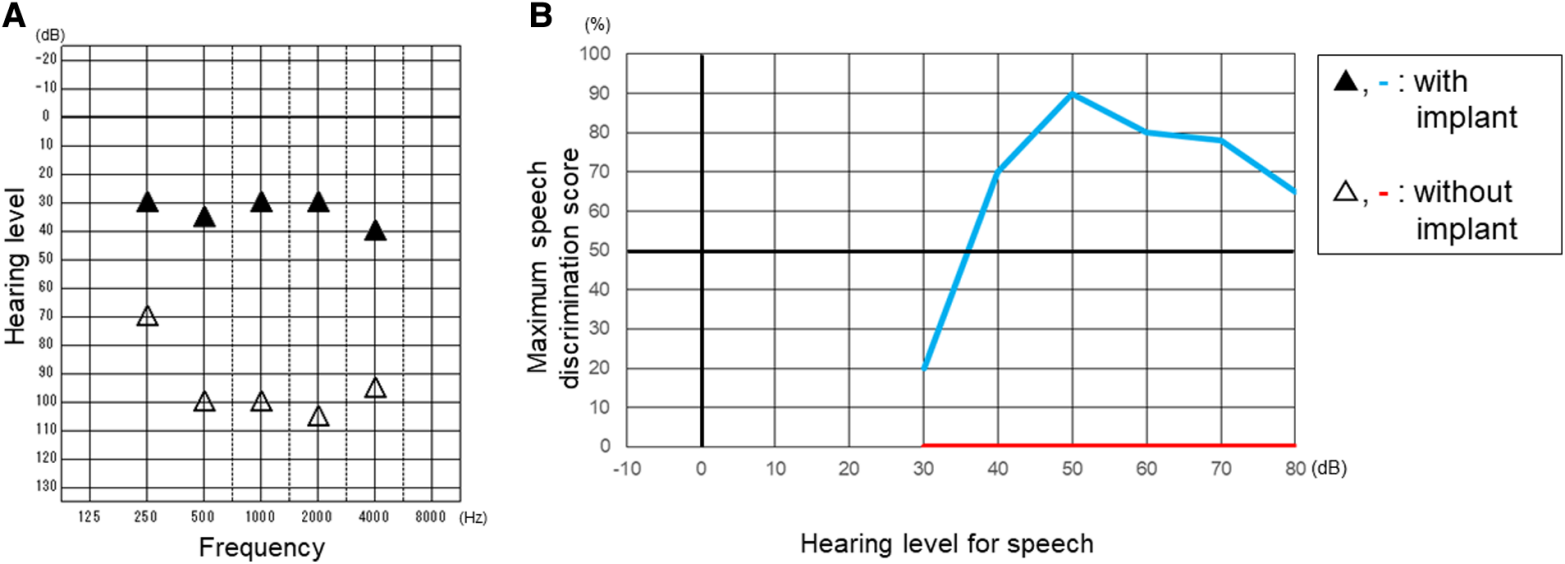
Play audiometry (**A**) and speech audiometry (**B**) after cochlear implantation. Maximum speech discrimination score was evaluated by using a table of meaningless monosyllables of 67-S proposed by the Japan Audiological Society. Black triangle and light blue line indicate the results with cochlear implant. White triangle and red line indicate the results without cochlear implant.

We applied a cochlear implant to the left ear. As preoperative evaluation by CT (Figure 1E) and MRI (Figure 1F) showed no ossification or fibrosis in the cochlea, a slim modiolar electrode was selected for the hearing preservation strategy. A shape memory electrode stored in the sheath (CI532, Cochlear Ltd.) was inserted through the round window, then the electrode was inserted along the inner wall of scala tympani to a sufficient depth without any resistance on the 1065th day after onset of KD (Figure 3). There were no fibrosis or cochlear lumen obstruction restraining simple cochlear implantation, as in the post-meningitis cases. All channels were inserted inside the cochlea and evoked compound action potentials in intraoperative neural response telemetry test were confirmed. After the cochlear implantation, his auditory communication skills dramatically improved and his mental status stabilized. One year after the surgery, a play audiometry test with the cochlear implant showed marked reduction in threshold at all frequencies compared with that without cochlear implant (Figure 4A). Speech audiometry showed 90% of maximum speech discrimination score with the cochlear implant, despite the results being completely indistinguishable without the cochlear implant (Figure 4B). We are recommending cochlear implantation to the other side considering advantages in young children of bilateral stimulation regarding speech understanding in noise, sound source localization and speech intelligibility (15).

## 3. Discussion

SNHL associated with KD is generally reported to be temporary with acute or subacute onset and is often reversible. This patient's course provides two important clinical suggestions: First, SNHL associated with KD could progress over the long term and result in severe hearing loss. Second, efficacy of cochlear implantation for bilateral severe SNHL associated with KD was demonstrated in this patient.

SNHL associated with KD could progress over a long term and result in severe hearing loss. SNHL is associated with about 30% of patients with KD and 73% of those patients develop bilateral hearing loss (3, 5–7). SNHL usually occurs during acute or subacute phases and is reversible (5, 7). A recent prospective study from Japan reported a low prevalence of SNHL, and routine hearing tests were said to be unnecessary (8). However, the routine schedule for hearing checkups included only two occasions and both within acute and subacute phases. Although there is even a report showing no significant difference in the incidence of hearing loss between KD and non-KD patients, the authors suggested that there might be subclinical cases that would not be captured in healthcare administrative data because children diagnosed with KD are not typically referred for audiological testing as part of their initial or follow-up surveillance in their region. They also suggested enhanced audiological surveillance among KD patients to promote earlier detection (16). As these studies show, the main part of cases of sensorineural hearing loss associated with KD is so transient and mild that even the parents do not notice it (17). Alves et al. reported that 11.3% of cases of SNHL associated with KD continued to have hearing loss six months after the first assessment (3), suggesting the importance of longer-term hearing follow up.

Development of severe hearing loss also seems to be rare in SNHL associated with KD (10–12). Etiologically, SNHL association with KD occurring in acute or subacute phases and often being reversible is perhaps related to administration of aspirin. Sundel et al. were the first group to suggest the potential role of aspirin ototoxicity because hypoalbuminemia caused by acute inflammation increases the level of free salicylate in the serum (18). However, Knott et al. noted that there was no statistically significant increase in risk of SNHL associated with elevated levels of aspirin in patients with KD (5). The involvement of aspirin into the pathogenesis of SNHL in KD thus remains controversial. Ototoxicity of cyclosporine is also controversial. Recent studies concluded that cyclosporine did not obviously increase the risk of sensorineural hearing loss (19, 20). Some studies suggested that because cyclosporine causes temporary SNHL in a dose-dependent manner, the concentration of cyclosporin in blood should be carefully monitored (19). Present patient was treated with acceptable dose of cyclosporine for a short term and blood concentrations of cyclosporin were confirmed to be within therapeutic range but not ototoxic levels.

Until now, gene mutations or variants responsible for SNHL associated with KD have not been identified, although some relating to coronary artery aneurysms have been reported (21). In our patient, although no inspectable gene variants relating to hearing loss were identified, not all previously reported gene variants were analyzed. Spread of genetic testing has accumulated increase evidence about gene mutations and variants causing hearing loss (22). The current case could provide genetic information about SNHL associated with KD. Physiopathologically, KD is an acute necrotizing systemic vasculitis of the medium- and small-sized arteries (1, 4). Vasculitis occurring at the vasa nervorum and perineural vessels may cause ischemic or osmotic changes in the inner ear hair cells, stria vascularis and acoustic nerve (6, 14), resulting in persistent SNHL associated with KD. Takeda et al. suggested that the small vessel abnormalities secondary to KD-induced vasculitis and intense immune activation would lead to damaged cochlear neurosensory cells (23). Signs of high and extended inflammatory activity, such as thrombocytosis, prolonged anemia and elevated erythrocyte sedimentation rate, were suggested to have significant association with SNHL in KD (3, 17). Interestingly, Cashman et al. recently demonstrated in an animal model that Lassa fever, which is caused by infection with Lassa virus, developed SNHL through a mechanism similar to autoimmune vasculitis. Elevation of some pro-inflammatory cytokines, including interleukin-1β, interleukin-6 and tumor necrosis factor-α, remained above baseline after clearance of the virus and were involved in the development of SNHL (24). These pro-inflammatory cytokines are considered to be potential biomarkers of KD (25). In our patient, high and persistent inflammatory responses including the elevation of WBC count and CRP, anemia and thrombocytosis were observed during the first onset and relapse of KD. Mild anemia and thrombocytosis were persistent during progression of SNHL after the relapse of KD. Thus, intensive immune reaction followed by vasculitis would therefore be a reasonable pathogenesis of persistent and progressive SNHL associated with KD. Kawata et al. suggested the importance of prevention of progressive systemic vasculitis by early initiation of treatment, which might have contributed to lower incidence of hearing loss (8). Future research about the etiology and development of biomarkers would provide further information predicting the prognosis of SNHL associated with KD. Although we could not perform to present patient, intratympanic injections of steroids is an accepted additional therapy in SNHL cases (26, 27). Local anti-inflammatory agent would be a reasonable and effective therapeutic option for SNHL associated with KD, if patient is acceptable.

Cochlear implantation could be effective for SNHL associated with KD. In terms of long-term outcomes of cochlear implantation to SNHL caused by autoimmune vasculitis, Bacciu et al. reported about Cogan syndrome, which is systemic vasculitis characterized by inflammation of the eyes and inner ears, and manifesting as interstitial keratitis and audiovestibulary dysfunction (28). Cochlear implantation improved word and sentence recognition scores to more than 90% at one and five years after implantation in all 12 patients with Cogan syndrome included in their study (29). An increasing number of case reports have demonstrated the efficacy of cochlear implantation in severe SNHL associated with other types of systemic vasculitis, such as antineutrophil cytoplasmic antibody (ANCA) associated vasculitis (30–32). Conversely, poor results of cochlear implantation for SNHL associated with ANCA associated vasculitis have also been reported (33, 34). Some systemic vasculitis, such as polyarteritis nodosa and ANCA-associated vasculitis causes labyrinthitis ossificans, a reactive response to the inflammatory vasculitis, determined by the formation of pathological fibrous tissue or new-bone formation in the inner ear (30, 34). Labyrinthitis ossificans causes closure of round window and narrowing of the basal turn of the cochlea, and results in poor auditory prognosis after cochlear implantation (34). Our patient showed no labyrinthine ossifications in the cochlea by both of imaging tests and intraoperative findings, and resulted in good auditory prognosis. Vasculitis of the inner ear, especially in the inner ear stria vascularis, which is reported to plays an important role in autoimmune inner ear diseases (35), will be the promising pathogenesis. And this hypothesis has reconfirmed by a good prognosis after cochlear implantation. With consideration of clinical settings, such as diagnosis, age, duration between onset and operation, or preoperative labyrinthine calcification, cochlear implantation could be an effective therapeutic option for bilateral severe SNHL caused by systemic vasculitis including KD. Because the efficacy of cochlear implantation varies with the clinical setting and it is important to accumulate evidence for each disease. Takeda et al. reported for the first time the impact of cochlear implantation in a case of 1-year-old boy who developed severe SNHL associated with KD (23). In this case report, we further reported the detailed clinical course of prognosis in KD in accordance with the Takeda's report. Taken together, these reports revealed the efficacy of cochlear implantation to SNHL associated with KD. One of the limitations of this report is that we could not rule out auditory neuropathy spectrum disorder, which is reported to have optimal outcome by cochlear implantation (36), because we did not perform otoacoustic emission test. Even so, present patient will have enough value and information to suggest the pathogenesis of SNHL associated by systemic vasculitis.

In conclusion, in our patient, SNHL associated with KD progressed over more than two years. This case suggests the importance of long-term auditory follow up among patients with KD, especially for children during cognitive and speech development. Cochlear implantation could be an effective therapeutic option for patients with SNHL associated with KD. Present report would provide the valuable evidence about the incidence, outcome and treatment for hearing loss associated with Kawasaki disease that have still not been well-established.

## Data Availability

The raw data supporting the conclusions of this article will be made available by the authors, without undue reservation.
