# Supplementary material for: Case report: Cochlear implantation was effective for progressive bilateral severe hearing loss associated with Kawasaki disease
Source: Front Pediatr. 2023 Aug 10;11:1199240. doi: 10.3389/fped.2023.1199240 (PMC10448821; doi:10.3389/fped.2023.1199240)
Supplement: Supplementary file 1 [file Datasheet1.pdf]

Supplementary Material

Case Report: Cochlear implantation was effective for progressive bilateral severe hearing loss associated with Kawasaki disease

Daichi Murakami[1†], Takahito Kimura[1†], Masamitsu Kono[1], Akihiro Sakai[1,2], Tomohiro Suenaga[3], Masanobu Hiraoka[1], Hideki Sakatani[1], Makiko Ohtani[1], Hiroyuki Suzuki[3, 4], Daisuke Tokuhara[3], Muneki Hotomi[1*]

[1]Department of Otorhinolaryngology-Head and Neck Surgery, Wakayama Medical University, Wakayama, Japan.

[2]Sakai ENT clinic, Kinokawa, Japan.

[3]Department of Pediatrics, Wakayama Medical University, Wakayama, Japan.

[4]Department of Pediatrics, Wakayama Tsukushi Medical and Welfare center, Iwade, Japan.

† DM and TK contributed equally to this work and are co-first authors.

*** Correspondence:** Muneki Hotomi: mhotomi@wakayama-med.ac.jp.

1 Supplementary Data: Perspective of the patient and his guardian

“At first, about 150 days after the development of Kawasaki disease, my hearing loss was pointed out at my daycare center. I could not speak in a whisper and could not hear music, even when using earphones. I began using hearing aids, but could not hear the TV unless the volume was turned up very loud. After recurrence of Kawasaki disease, I could no longer react to sound or hold a conversation. I did not talk much anymore and could no longer keep up with my elementary school classes. I was not sure whether I should have cochlear implantation, but after the operation, I gradually began to hear sounds and conversations around me. I am glad that I can now hear birdsong and the sound of rain.”
